# Vemurafenib- and Cobimetinib-Associated Drug Reaction With Eosinophilia and Systemic Symptoms in a Patient With Metastatic Melanoma

**DOI:** 10.7759/cureus.44462

**Published:** 2023-08-31

**Authors:** Miriam Al-Saedy, Salsabeal Al-Saedy, Chad Rieck

**Affiliations:** 1 Dermatology, Elson S. Floyd College of Medicine, Spokane, USA; 2 Medicine, Elson S. Floyd College of Medicine, Spokane, USA; 3 Internal Medicine, Providence Regional Medical Center, Washington State University, Elson S. Floyd College of Medicine, Everett, USA

**Keywords:** dress, clinical research, drug response, biologics, immunodermatology, immunotherapy, drug reaction, complex dermatology, medical dermatology, general dermatology

## Abstract

Drug reaction with eosinophilia and systemic symptoms (DRESS) is a severe hypersensitivity reaction associated with drug exposure. Recognizing signs of DRESS and stopping the offending agent is essential for proper treatment. In this case report, we present an interesting case of DRESS following the recent initiation of vemurafenib and cobimetinib for the treatment of metastatic melanoma in a patient who previously had been on pembrolizumab without adverse skin reactions. In this case report, we highlight the ambiguity of using the Registry of Severe Cutaneous Adverse Reactions (RegiSCAR) scoring criteria in the hospital setting for recognizing DRESS in patients with toxic epidermal necrolysis (TEN)-type presentation of DRESS.

## Introduction

Drug reaction with eosinophilia and systemic symptoms (DRESS) is a severe and potentially life-threatening dermatological emergency, that is a hypersensitivity reaction associated with drug exposure. No specific diagnostic test currently exists for DRESS. Rather, it is usually characterized by the Registry of Severe Cutaneous Adverse Reactions (RegiSCAR) criteria [1], which include three main requirements of acute rash, hospitalization, and suspicion of a drug-related reaction. DRESS is known to mimic Stevens-Johnson syndrome (SJS)/toxic epidermal necrolysis (TEN) and other drug-induced severe cutaneous adverse reactions (SCARs) which can lead to a delay in diagnosis and proper treatment [2]. The diagnosis of DRESS requires clinical suspicion and exclusion of other autoimmune, neoplastic, inflammatory, and infectious causes including the exclusion of other drug-induced adverse cutaneous reactions [3]. Recognizing signs of DRESS and stopping the offending agent is essential for proper treatment. In this case report, we present an interesting case of DRESS following the recent initiation of vemurafenib and cobimetinib for the treatment of metastatic melanoma in a patient who previously had been on pembrolizumab without adverse skin reactions.

## Case presentation

A 61-year-old Caucasian male with a past medical history of metastatic melanoma with BRAFV600E mutation presented to the hospital with a new-onset generalized body rash 12 days following initiation of vemurafenib and cobimetinib. The patient began 960 mg of vemurafenib and 60 mg (once every day 1 and day 21 of each 28-day cycle) of cobimetinib. After three days he began noticing lip swelling and irritated itchy lips and face with minimal improvement on 1% hydrocortisone. He then experienced a full-body rash eruption beginning with pruritus in the mouth, hands, and spreading to the legs and upper extremities and face. On day 10, the patient then began to experience a full-body generalized rash, difficulty eating, feeling lightheaded, and collapsed three times with temporary loss of consciousness. On day 11, he stopped his oral chemotherapy medications and began 200 mg of oral prednisone. On hospital admission the next day, the patient still had a generalized rash, endorsed lightheadedness, difficulty eating with pain with swallowing, and ocular vision changes (gray spots in vision).

**Figure 1:**
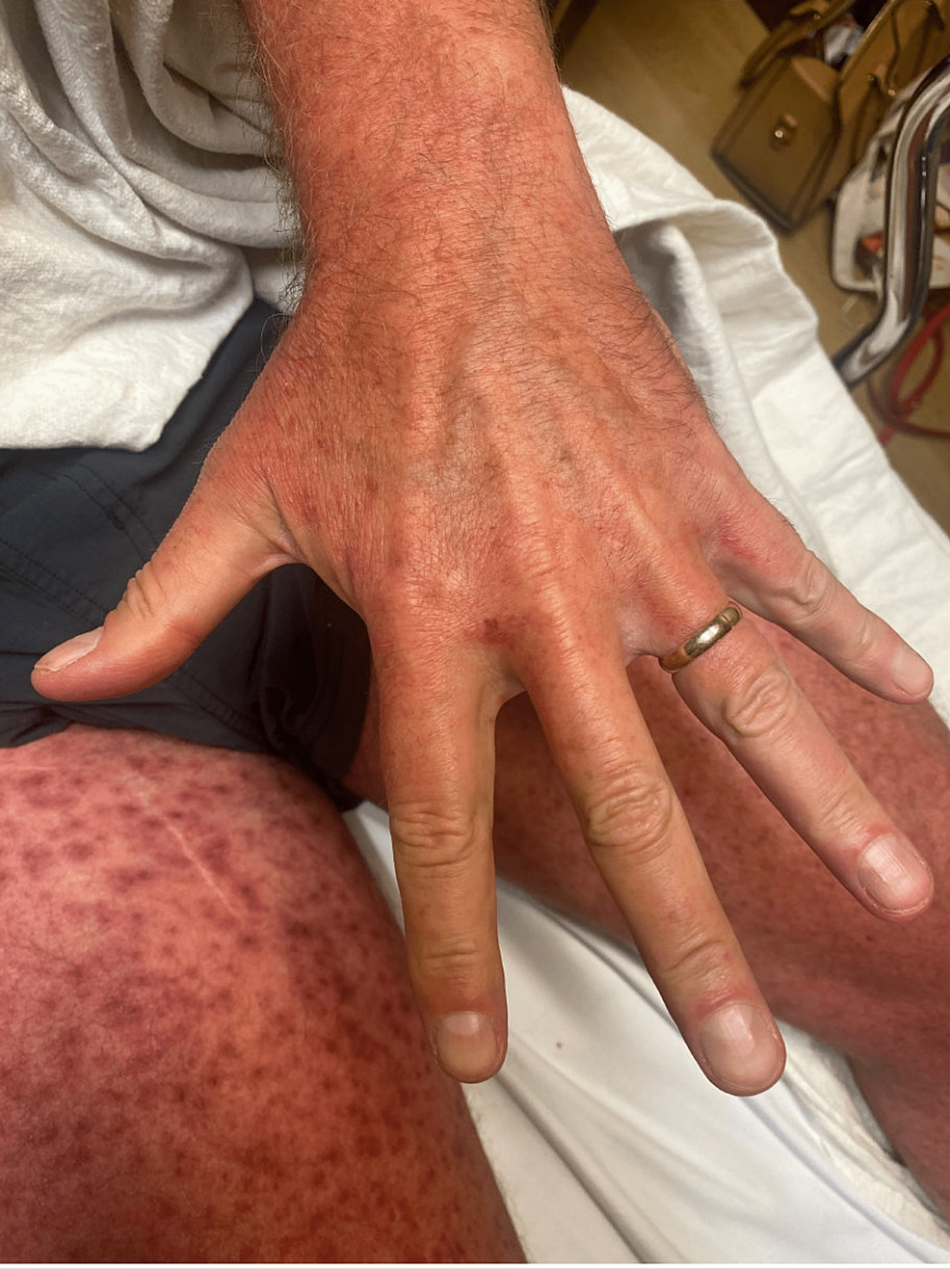
Hand with erythematous rash

**Figure 2:**
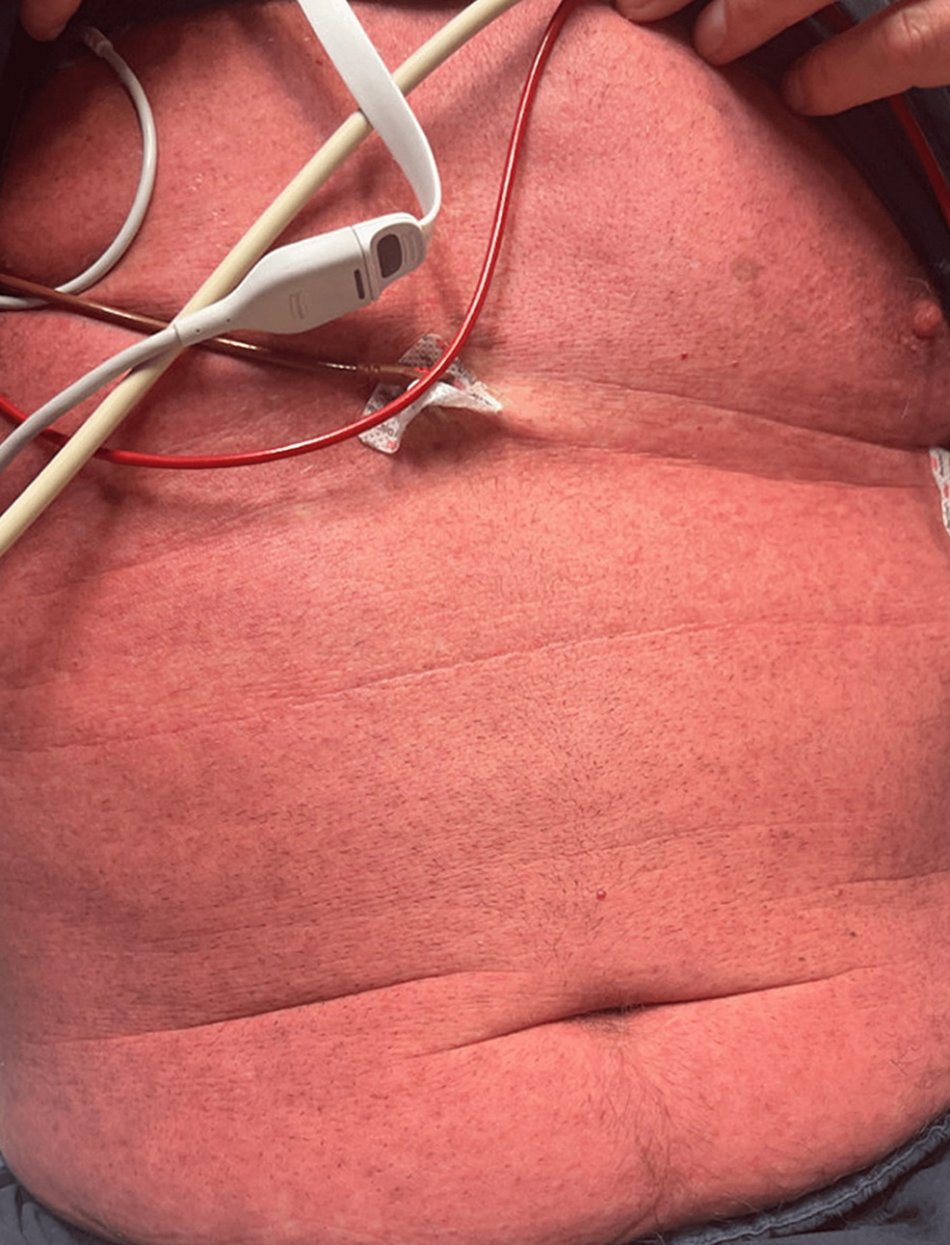
Abdomen with erythematous morbilliform-like rash

**Figure 3:**
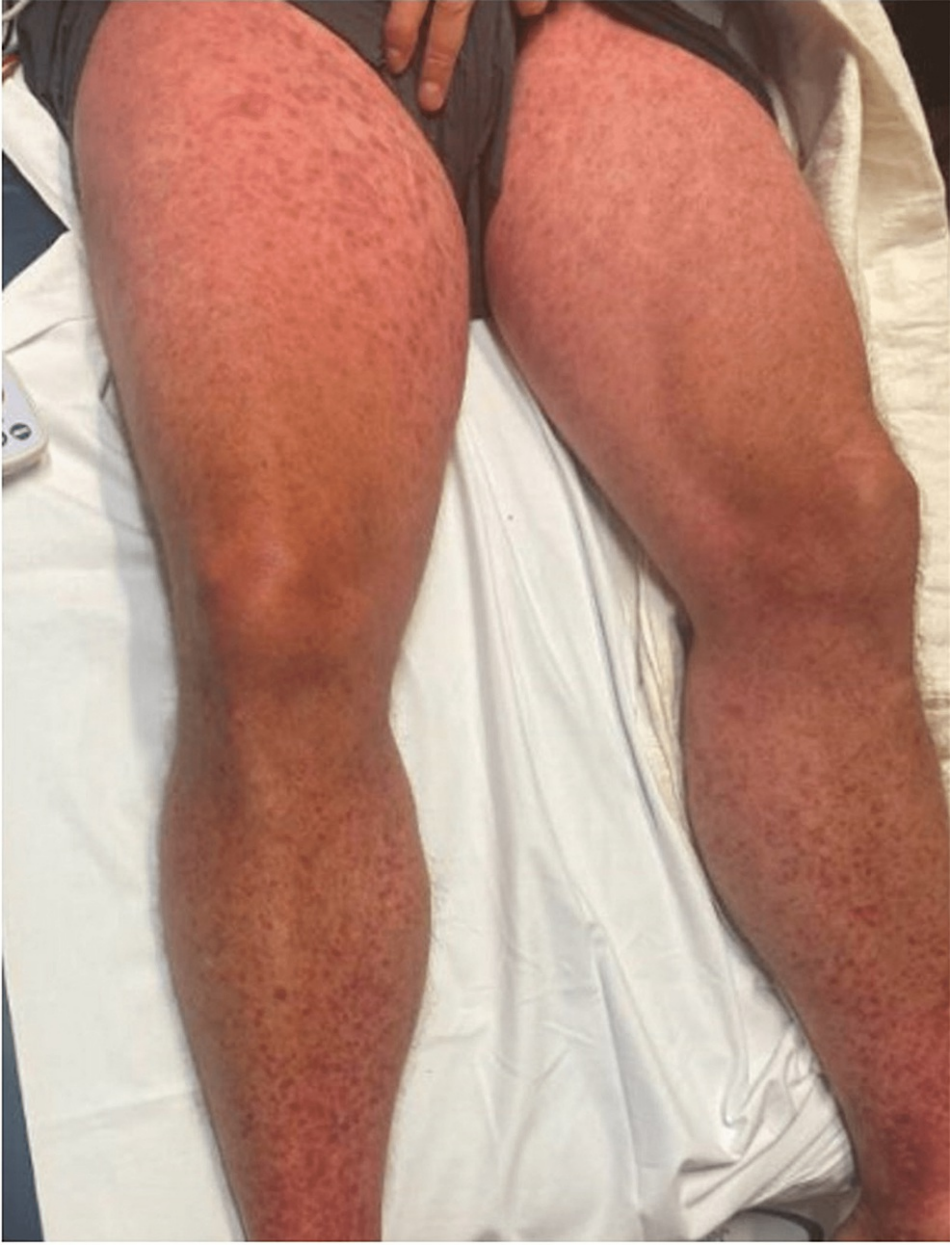
Lower extremities with macular erythematous rash

The patient has no history of allergies to medications, soaps, or detergents, and no personal or family history of eczema or psoriasis. The patient has never had a rash like this before. His vitals were stable and afebrile. He was otherwise in no acute distress. On physical examination, the patient had a bright red generalized morbilliform blanchable macular rash without desquamation, superficial scale, or bullae and a negative Nikolsky sign (Figures 1-3).

He had morbilliform/purpura-like eruption in the lower extremities, lips irritated without mucosal ulcers, posterior oropharynx with tonsillar erythema and exudate present with a single ulcerative lesion on the left tonsil 8 mm in size, with irregular borders, and conjunctival injection present bilaterally. Lab findings are shown in Table 1. Lab findings were significant for thrombocytopenia, lymphopenia, and elevated creatinine. 

**Table 1:** Laboratory Findings WBC, white blood cells; AST, aspartate transaminase; CRP, C-reactive protein; BNP, brain natriuretic peptide; ANA, antinuclear antibodies; ANCA, anti-neutrophil cytoplasmic antibody.

Laboratory findings	
WBC	3.7 k/uL
Platelets	96 k/uL
Creatinine	2.23 mg/dL
AST	49 U/L
Lactate	2.0 mmol/L
Fibrinogen	301 mg/dL
CRP	22.5 mg/dL
BNP	25 pg/mL
Hepatitis A virus	Nonreactive
Hepatitis B virus	Nonreactive
Hepatitis C virus	Nonreactive
HIV	Nonreactive
ANA/ANCA	Negative
C3/C4 complement	141/38 mg/dL
Troponin I	80 ng/L

**Figure 4:**
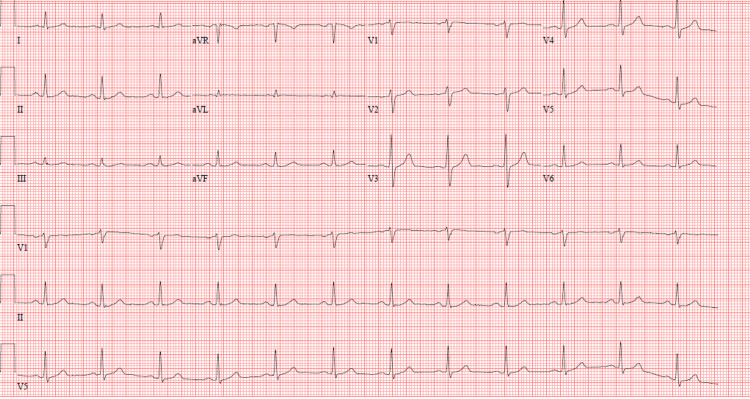
Twelve-lead electrocardiogram

A syncope workup including EKG with normal sinus rhythm (Figure 4), brain natriuretic peptide (BNP), echocardiography, and orthostatic blood pressure was completed, which did not suggest cardiac cause of his history of reported syncope.

Exclusion of hepatitis A virus (HAV), hepatitis B virus (HBV), hepatitis C virus (HCV), and HIV was completed. A chest X-ray was completed to rule out mycoplasma/atypical pneumonia. The antinuclear antibody (ANA)/anti-neutrophil cytoplasmic antibody (ANCA) was negative, C3/C4 complement levels were normal, and blood cultures were negative.

DRESS was considered due to the patient recently initiating a new medication approximately 12 days prior to presentation. The patient had a “probable” score of 5 per RegiSCAR criteria due to a history of thrombocytopenia, eosinophilia based on skin biopsy, greater than 50% rash body surface area, purpura-like lower extremity rash with mild edema, internal organ involvement (ocular, oropharyngeal), peripheral blood smear showing toxic changes of mildly left shifted neutrophils suggestive of a reactive etiology, and alternative diagnoses excluded. Due to the likelihood of DRESS vs. less likely vasculitis, a punch biopsy of the right thigh was performed. The patient was started on 125 mg IV methylprednisolone. His troponin was elevated at 80 and trended down to 40 with IV steroid treatment. His creatinine has trended down to 1.52. On day 3 of IV steroids the patient began to have improvement of conjunctival injection and ocular vision changes, no lightheadedness, and a duskier appearance of the generalized rash with desquamation. Also, the patient experienced improvement in eating with decreased pain.

The punch biopsy later confirmed that the patient had apoptotic and dyskeratotic keratinocytes and superficial perivascular chronic inflammation with eosinophils suggestive of DRESS.

## Discussion

Herein we report an unusual case of a 61-year-old male with a history of metastatic melanoma who presented with TEN-appearing DRESS with oropharyngeal and ocular involvement 12 days following initiation of vemurafenib and cobimetinib. The patient experienced rapid resolution of the rash and improvement of systemic symptoms with desquamation of the rash after both discontinuing vemurafenib and three days of IV steroid treatment. DRESS can be life-threatening and a delay in diagnosis is associated with worse outcomes. He was previously on prior PD-1 (programmed cell death protein 1) inhibitor immunotherapy, pembrolizumab, for 2.5 years for metastatic melanoma without similar rash or systemic symptoms. Complete blood count (CBC) did not show eosinophilia due to the patient taking steroids, but the patient did have eosinophils on skin biopsy which can suggest a score of 6 per RegiSCAR and a definitive diagnosis of DRESS. The patient was given a one-month 60-40-20-10 mg steroid taper. Interestingly, in RegiSCAR, a skin biopsy suggesting DRESS is scored 0 points. There have been reports of patients with DRESS not meeting diagnostic criteria per RegiSCAR but were diagnosed clinically [4]. Furthermore, the skin biopsy was consistent with severe drug reactions. Of note, the histopathology of DRESS has not been well specified and is typically labeled as “EM-like, spongiotic, lichenoid, or toxic epidermal necrolysis-like” [5]. The combination of BRAF and mitogen-activated protein kinase kinase (MEK) inhibitors has become the standard of care in metastatic melanomas with the BRAFV600E mutation. Additionally, there have been reports of cases of vemurafenib-inducing DRESS with subsequent melanoma regression [6]. Interestingly, one of the cases is a 65-year-old male who was taking 960 mg BID of vemurafenib for three weeks who then developed DRESS, and upon discontinuation of the drug, 10 months later, had regression of melanoma without any other treatment [7]. Increased awareness regarding the association between DRESS and vemurafenib will help with diagnosing future cases in avoiding the morbidity of delayed diagnosis.

## Conclusions

In conclusion, DRESS can be life-threatening and a delay in diagnosis is associated with worse outcomes. The patient was previously on prior PD-1 inhibitor immunotherapy, pembrolizumab, for 2.5 years for metastatic melanoma without similar rash or systemic symptoms. The patient presented with a TEN-type picture of DRESS which made RegiSCAR scoring criteria of DRESS challenging. Physical and systemic symptoms improved following both discontinuation of responsible drugs and treatment with IV methylprednisolone. Future research assessing the utility of the RegiSCAR scoring criteria in monitoring for DRESS with TEN-type presentation is warranted to guide proper recognition and treatment, especially for non-dermatology-trained practitioners.
